# Beyond the learning curve: length of global health electives

**DOI:** 10.5116/ijme.57c1.4e07

**Published:** 2016-09-11

**Authors:** Elizabeth M. Keating, Heather Lukolyo, Chris A. Rees, Eric J. Dziuban, Margaret G. Ferris, Gordon E. Schutze, Stephanie A. Marton

**Affiliations:** 1 Department of Pediatrics, Baylor College of Medicine, Houston, TX, USA; 2 Department of Neuroscience, Baylor College of Medicine, Houston, TX, USA

**Keywords:** Learning curve, global health electives

## Introduction

Medical students and residents from resourced regions are increasingly seeking clinical rotations in resource-limited settings abroad. This interest has been matched by increased opportunities in many residency programs in the United States for residents to participate in global health electives (GHEs). Global health electives provide learners with opportunities to practice and learn within global health settings. This enhances their knowledge of local diseases and disease processes, leads to improved physical exam skills with decreased reliance on labs and other testing, and demonstrates the importance of communication across cultures and languages. In addition, studies have shown that short-term learners (STLs) who participate in GHEs are more likely to practice primary care medicine, obtain public health degrees, and practice medicine amongst underserved populations in their home countries.^1^

In 1999, the American Academy of Pediatrics (AAP) recommended that GHEs last a minimum of four weeks to allow learners to assimilate into the culture and maximize their time abroad.^2^ Currently, GHEs offered in pediatric residency programs range from three to eight weeks in length.^3^ To our knowledge there have been no discussions about the reasons for the recommended duration of four weeks for GHEs. We aimed to describe how clinical productivity of STLs varies by consecutive working days on a GHE.

### Evaluating global health rotation length

The Baylor International Pediatric AIDS Initiative at Texas Children’s Hospital clinical network is the largest network of pediatric HIV clinics worldwide.^4^ Short-term learners from institutions in North America have been completing GHEs at the Baylor College of Medicine Bristol-Myers Squibb Children’s Clinical Centre of Excellence - Swaziland (Baylor-Swaziland) since 2006. In 2010, 26 STLs completed GHEs at Baylor-Swaziland, including medical residents, medical students, and non-clinical learners. Short-term learners spent two to four weeks with Baylor-Swaziland. The electronic medical records in Swaziland from 2010 were reviewed to assess the total number of patient encounters completed by both resident or fellow STLs and clinical preceptors per day.

Based on the number of patient encounters per medical resident learner per day, there was a sequential increase in the number of patient encounters with more sequential workdays of the rotation. The number of patient encounters per day did not vary by level of STL training, with residents in their second, third, and fourth years of residency completing similar numbers of patient encounters per day. The number of patients seen by two different expatriate clinical preceptors during a one-month period was not affected by mentoring STLs.

From our observations, STLs completed more patient encounters per day over time. This suggests that there is an inherent learning curve during a GHE. While the AAP currently recommends that GHEs last four weeks, longer rotations may offer STLs additional time to contribute to their site in meaningful ways. Studies assessing host preceptors’ perceptions of STLs have shown a preference for rotations lasting four to six weeks.^5^ Other studies have suggested an even longer elective length between two and three months,^6^ which may offer more opportunities to master a range of competencies in a global health setting.

The learning curve we observed is likely due to a combination of operational, clinical, and cultural-linguistic factors. Operational factors include becoming familiar with the electronic medical record, clinic flow, and clinic processes. The concept of a learning curve in residency has been demonstrated in several fields.^7^ However, in a setting such as Swaziland, there are many clinical factors that STLs may not have encountered before. These include unfamiliarity with diseases that are less commonly encountered in the home setting, not being well versed in local or international clinical guidelines, not knowing the available medications, and inexperience working within resource limitations. Particularly in the Baylor-Swaziland clinic setting, management of pediatric HIV is unfamiliar to most STLs. Short-term learners are also likely to encounter some degree of cultural adjustment or emotional challenges in the new environment which may impact clinical productivity. In addition, language barriers and the need to use an interpreter during clinical encounters likely hinders productivity.

Though our findings suggest that longer rotations may allow learners to get over the initial learning curve, there are many barriers to increasing rotation length for trainees. These include providing adequate call coverage at the home institution, funding of resident salaries when they are away, and restrictions by the Accreditation Council of Graduate Medical Education on time away.^8^ It would be ideal for programs to have the flexibility to have call- and continuity clinic-free months without jeopardizing accreditation or causing burdens on fellow residents. Furthermore, some residency programs require vacation to be taken during GHEs, further shortening actual time spent at host clinical sites. Between vacation, travel time, weekends, and possible public holidays, the actual clinical experience may be as short as ten working days or less. According to a recent survey of residents, 57% would not be willing to give up vacation time during international electives.^9^ Nevertheless, requiring residents to do so may have the favorable effect of selecting for those residents who are most dedicated to or interested in global health, which has been cited by international host preceptors as a desirable trait of STLs completing GHEs at their sites.^10^

Interestingly, the number of patient encounters per day did not vary by medical resident training level. This supports the argument that everyone, regardless of medical resident year of training, encounters an initial learning curve. Reassuringly, STLs in our study did not appear to hinder clinical preceptor productivity in terms of patient encounters per day. This is in contrast to other reports in which there was perceived efficiency burden on clinical preceptors of STLs.^10^ These differing findings may be explained by the measure of clinical efficiency (i.e. self-perceived versus directly measured). Additionally, STL mentoring tasks may detract from other non-clinical administrative roles that preceptors have in their clinics. Finally, the lack of hindrance on productivity could be because the host preceptors at Baylor-Swaziland were expatriate clinicians who may have had greater understanding of STLs’ capabilities and training background.

## Conclusions

There is an inherent learning curve associated with completing an elective at a global health site. Short-term learners completing GHEs should plan to stay long enough to get beyond the learning curve to maximize both clinical productivity and their own learning. Since productivity levels were similar across medical resident training levels, sites are encouraged to host STLs at various levels of training.

### Conflict Of Interest

There is no conflict of interest that might bias the outcomes of this paper.
